# Detection and analysis of disease-associated single nucleotide polymorphism influencing post-translational modification

**DOI:** 10.1186/1755-8794-8-S2-S7

**Published:** 2015-05-29

**Authors:** Yul Kim, Chiyong Kang, Bumki Min, Gwan-Su Yi

**Affiliations:** 1 Department of Bio and Brain Engineering, KAIST, Daejeon, South Korea

**Keywords:** Post-translational modification (PTM), single nucleotide polymorphism (SNP), genome-wide association study (GWAS), PTM-SNP

## Abstract

Post-translational modification (PTM) plays a crucial role in biological functions and corresponding disease developments. Discovering disease-associated non-synonymous SNPs (nsSNPs) altering PTM sites can help to estimate the various PTM candidates involved in diseases, therefore, an integrated analysis between SNPs, PTMs and diseases is necessary. However, only a few types of PTMs affected by nsSNPs have been studied without considering disease-association until now. In this study, we developed a new database called PTM-SNP which contains a comprehensive collection of human nsSNPs that affect PTM sites, together with disease information. Total 179,325 PTM-SNPs were collected by aligning missense SNPs and stop-gain SNPs on PTM sites (position 0) or their flanking region (position -7 to 7). Disease-associated SNPs from GWAS catalogs were also matched with detected PTM-SNP to find disease associated PTM-SNPs. Our result shows PTM-SNPs are highly associated with diseases, compared with other nsSNP sites and functional classes including near gene, intron and so on. PTM-SNP can provide an insight about discovering important PTMs involved in the diseases easily through the web site. PTM-SNP is freely available at http://gcode.kaist.ac.kr/ptmsnp.

## Introduction

Since the advance of next-generation sequencing (NGS) technologies, the number of single nucleotide polymorphisms (SNPs) keeps increasing precipitously and it becomes crucial issue to discovering functional implication of SNPs on the biological process and complex diseases. Especially, non-synonymous SNPs (nsSNPs), including missense SNPs that provoke amino acid mutation and stop-gain SNPs that terminate the peptide synthesis by generating stop codon, are thought to play key roles in causing diseases by changing the protein structure and functions. It is estimated that nsSNPs cover more than half of the disease-causing mutation in the Human Gene Mutation Database [1].

In this regard, several efforts have been made to elucidate the effect of nsSNPs on diseases recently. For example, CanPredict [2] and Diseasome [3] tried to predict the effect of nsSNPs on disease using SIFT [4] or PolyPhen [5]. However, although previous studies predicted disease-associated nsSNPs, related biological process have not been provided and hence have limits to understand detailed disease mechanisms. Therefore, an additional in-depth study about correlation between the location of nsSNPs and functional site of proteins is necessary. David et al. reported that disease-associated nsSNPs in the protein interaction interface affect the protein stability and prohibit protein interactions [6]. Alternatively, post-translational modification (PTMs) sites are another important functional parts of the protein and nsSNP influencing PTM sites should be considered.

Post-translational modification plays essential roles in the most biological pathways and disruption of PTM sites is known as the major cause of diseases [7]. Radivojac, et al. predicted phosphorylation-related variants from disease-associated variants and found that phosphorylation binding site disrupting variants are correlated with somatic cancer mutations [8]. Recently, several previous studies [9-11] detected PTM-related nsSNPs or protein sequence variations. Yang et al. [9] collected 15,738 experimental phosphorylation binding sites and found 1,515 coding-region SNPs in the flanking phosphorylation binding sites with position -7 to +7. Ryu et al. [10] collected 33,651 protein-sequence variations from the SwissVariant database and predicted the effects of variants on phosphorylation binding sites using a phosphorylation-related variant prediction tool. Ren et al. [11] collected 91,797 nsSNPs from dbSNP Build 130 and mapped nsSNPs onto mRNA/Protein sequences from RefSeq Build 31 [12]. They collected 64,035 phosphorylation-related nsSNPs in 5 categories using GPS 2.0 [13], a phosphorylation binding site prediction tool. However, previous studies are tend to focus on phosphorylation even there exists other kinds of PTMs like ubiquitination that is related in protein degradation [14]. They also didn't consider the statistical significance with disease-association of PTM-SNPs with genome-wide association studies.

Here, we built a comprehensive PTM-SNP database containing more than 50 kinds of experimentally validated PTMs and predicted PTMs. While previous studies considered phosphorylation, PTM-SNP can examine multiple PTMs simultaneously. We also matched PTM-SNPs with various disease-associated SNPs based on GWAS to find disease-associated PTM-SNPs. The statistical significance values of the disease-association from GWAS are provided for accurate consideration of disease-association. We found that PTM-SNPs are highly correlated with diseases, compared with other SNPs in functional units. In addition, the result of the case study about PTM-SNP on type 2 diabetes shows our PTM-SNP provides an insight into unveiling important PTMs involved in causing diseases and finding novel disease markers. The web interface of PTM-SNP make easier to access our data and be conjugated as useful evidences in biomedical studies like drug development or predicting diagnosis and drug response.

## Materials and methods

### Collection of nsSNP data and PTM site data

We collected total 517,466 human nsSNPs from the NCBI dbSNP database Build 135 [15]. From 517,466 nsSNPs, 509,183 SNPs were missense SNPs and 14,878 SNPs were stop-gain (nonsense) SNPs. We linked the location of nsSNPs on protein sequences from Ref-Seq Build 37.3. The flanking peptide of nsSNP sites from -7 to +7 retrieved by using the SNP-MapLinkProtein table in dbSNP and defined as the SNP sequence.

Human PTM site data were retrieved from dbPTM3 [16], which includes 194,886 experimentally verified PTMs from MS/MS analysis based research articles and 10 external PTM-related resources. The dbPTM also provides 404,501 computationally predicted PTMs by the KinasePhos-like method based on hidden Markov models (HMMs). It includes 20 types of PTM sites predicted with the threshold of specificity 100% for minimizing false positive. Peptides of flanking PTM sites from -7 to +7, called the PTM site sequence, were retrieved by matching the PTM site with the protein sequence from UniProt [17].

### Detection of PTM-SNP

**Figure 1:**
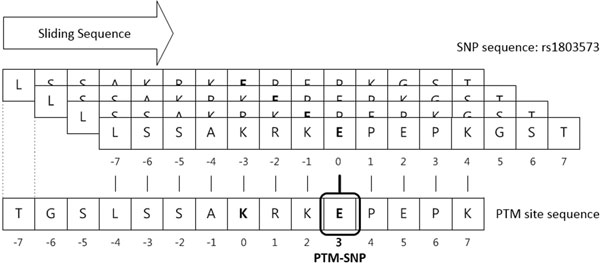
**Detection of a PTM-SNP by alignment of a PTM site sequence and a SNP site sequence**. Start from -7 to 7 residues of PTM site sequences, each residue is matched with nsSNP site of the SNP sequence by sliding one-by-one. From the best aligned position of SNP sequence, the location of PTM-SNP is identified as the location of the PTM site sequence that is aligned with the nsSNP.

PTM-SNPs were detected by aligning the SNP sequence with the PTM site sequence because not all sequences in UniProt and RefSeq are perfectly identical (end-to-end identical). Each residue in the SNP sequence is matched with the PTM site sequence by sliding one-by-one (Figure 1). The PTM-SNP is defined as the nsSNP's position of the best-aligned SNP sequence with the PTM site sequence. As Figure 1 illustrates, an SNP sequence of nsSNP, rs1803573, is aligned well with the +3 residue of the PTM site sequence. In few cases, multiple hits with diverse locations were detected due to sequence repeats. The European Molecular Biology Open Software Suite (EMBOSS) [18] Needle, a global pairwise sequence alignment tool, was applied to handle these exceptional cases.

We also extracted and integrated PTM-SNPs from the public resource, PhosSNP 1.0. PhosSNP includes phosphorylation-associated nsSNP from computationally translating mRNA sequences. However, about 18% of SNPs in PhosSNP have not yet been defined as nsSNP by dbSNP Build 135, therefor, were removed in the integrating process.

### Disease-associated PTM-SNPs

From GWASs using SNP arrays and NGS techniques, huge numbers of statistically significant disease-associated SNPs have been identified recently. National Human Genome Research Institute (NHGRI) GWAS catalog have been provided 8,771 trait-associated SNPs with p-values < 1.0 × 10^-5 ^(Apr 02, 2013) [19]. The genetic association database (GAD) [20] contains 29,578 disease-associated SNPs and 23,671 disease-associated SNPs are also available from Genotypes and Phenotypes (dbGaP) [21]. To find a correlation between PTM-SNPs and diseases, we collected total 52,731 distinct disease-associated SNPs from NHGRI GWAS catalog, GAD and dbGaP after then matched these SNPs with our PTM-SNPs.

Furthermore, we analyzed type 2 diabetes (T2D) associated SNPs that were identified in our previous study [22] by using Wellcome Trust Case Control Consortium (WTCCC) datasets [23]. T2D-associated SNPs with p-values < 1.0 × 10^-5 ^were identified from quality controlled (QC) 409,656 SNPs, based on Cochran-Armitage trend test statistics using PLINK 1.07 [24]. QC is applied as a sample missing genotype rate of > 3%, a SNP missing genotype rate of > 1%, Hardy-Weinberg Equilibrium (HWE) p-value ≤ 10^-4^, and minor allele frequency (MAF) < 1%. Linkage disequilibrium pruning was leaved out to preserve the PTM-SNP candidates. T2D PTM-SNPs were detected by mapping T2D-associated SNPs and PTM-SNPs. Significantly associated T2D PTM-SNPs were further studied to interpret disease mechanisms with various biological information resources. Known disease genes are mainly collected from OMIM [25], GAD, and KEGG [26]. Drug targets are collected from DrugBank [27], PharmGKB Drug [28], and KEGG Drug.

## Result and disscussion

### Data Statistics

PTM-SNP were collected by integrating dbPTM-dbSNP matching and PhosSNP 1.0. Totally, distinct 179,325 PTM-SNPs were identified, consisting of 133,266 PTM-SNPs from dbPTM-dbSNP matching and 64,035 PTM-SNPs from PhosSNP. All PTM-SNPs were categorized into 3 groups based on the SNP position; PTM-SNPs on PTM site (position 0), PTM-SNPs flanking PTM sites (position -7 to 7), and stop-gain PTM-SNPs (Table 1).

**Table 1:** PTM-SNP statitsics based on SNP locations

Post-translational Modification	dbPTM-dbSNP matching		PhosSNP		PTM-SNP
	
	Experimental	Predicted	Experimental	Predicted	
On the PTM site	4,385	5,098	172	16,954	26,100

On the flank of the PTM site	52,249	76,654	1,836	59,340	163,254

Stop-gain affected PTM site	7,757	10,432	48	442	11,940

**Total**	60,380	88,181	2,004	6,4035	179,325

We also categorized PTM-SNPs based on PTM types. Table 2 shows the top 10 PTMs which have high PTM-SNP ratio (All PTMs are listed in the additional file 1). From the 599,382 PTM sites in dbPTM3, 48.45% were related with at least one PTM-SNP. Especially, the result represents that PTM types involved in the signal transduction, such as phosphorylation, ubiquitination, and acetylation-sites, have a greater proportion of PTM-SNPs than other PTM types. The proteolytic cleavage sites and disulfide bond-sites also have a higher proportion of PTM-SNP where they can change protein structure dramatically.

**Table 2:** Statistics of PTM Site based on PTM types with the Top 10 ratio

Post-translational Modification	Number of PTM sites	Number of PTM Sites of PTM-SNPs	Ratio
Disulfide bond	1,750	1,230	0.703

Ubiquitination	34,507	21,824	0.633

Proteolytic Cleavage	1,569	987	0.629

Acetylation	11,612	6,760	0.582

Phosphorylation	355,203	203,830	0.574

Sumorylation	1,180	673	0.570

S-nitrosylation	1,286	695	0.540

N-linked Glycosylation	65,121	21,215	0.326

Methylation	6,166	1,761	0.286

Sulfation	8,614	2,433	0.282

O-linked Glycosylation	91,678	21,335	0.233

Other PTMs	20,696	7,670	0.371

Total	599,382	290,413	0.485

### Correlation between PTM-SNP and Diseases

To elucidate the disease mechanism from PTM perspective, we matched PTM-SNPs with disease-associated SNPs. To estimate the importance of PTM-SNP on causing diseases, we compared the coverage rate of disease-associated SNPs between PTM-SNPs with other missense and stop-gain SNPs not related with PTM sites (Table 3). Total 281 disease-associated SNPs in NHGRI GWAS catalog was applied in the comparison and 186 of them were identified as PTM-SNPs. As a result, the coverage ratio of disease-associated PTM-SNP was more than four times than the ratio of non PTM-SNP. Although the coverage ratio is too low because the number of disease-associated SNPs are too small than the number of SNPs, p-values calculated by fisher's exact test shows our result is significantly associated with the disease.

**Table 3:** Statistics of disease-associated SNPs based on Functional Categories

Functional classification	Number of SNPs	Number of Disease-associated SNPs	Coverage Ratio	P-value
Missense & Stop-gain (PTM-SNP)	179,325	186	0.00114	2.72E-67

NearGene-5	744,086	251	0.00033	7.59E-13

UTR-3: MirSNP	414,510	151	0.00036	2.76E-10

UTR-3	513,249	170	0.00033	1.61E-08

NearGene-3	189,105	71	0.00037	2.54E-06

UTR-5	80,250	35	0.00043	3.33E-05

Cds-synon	312,479	100	0.00032	4.54E-05

Missense & Stop-gain (Non PTM-SNP)	361,401	95	0.00026	0.014

Intergenic	20,492,263	4,357	0.00021	0.136

Frameshift	30,578	5	0.00016	0.768

Intron	19,248,959	3,678	0.00019	0.999

**Total SNPs**	41,740,143	8,771	0.00021	-

Not only missense and stop-gain SNPs, we also compared different functional SNPs in dbSNP. Functional classes that contain more than 100 disease-associated SNPs were considered in comparison. In addition, 414,510 SNPs on miRNA-mRNA binding site were collected from MirSNP [29]. The result shows that SNPs near the gene, such as NearGene-3, MirSNP, NearGene-5 and UTR-3 were higher disease-associated proportion than SNPs in intron because they might affect protein functions by indirect way like regulating protein expressions. On the other hand SNPs around PTM site can give a potent influence on the protein and, as a result, the ratio of disease-associated PTM-SNP was superior to other functional classes. Therefore, we can conclude that PTM-SNPs are highly associated with diseases compared with both other nsSNPs and other functional sites.

Table 4 demonstrates the statistics of disease-associated SNPs based on PTM categories. Eight PTM types contains at least one disease-associated SNPs from the NHGRI GWAS catalog. The result shows phosphorylation is highly significant with disease association while it has a p-value lower than 0.05. Table 5 demonstrates the statistical analysis of trait or disease-associated PTM-SNPs (Whole statistics are listed in additional file 2). Disease-associated SNPs were collected from the NHGRI GWAS catalog, GAD, and dbGaP to match with PTM-SNPs. From the statistical analysis of disease-associated PTM-SNPs and disease-associated SNPs for top-ranked diseases, complex diseases, such as coronary heart disease and T2D are highly correlated with PTM-SNPs.

**Table 4:** Statistics of disease-associated SNPs based on PTMs

Post-translational Modification	Number of Disease Associated PTM-SNP	Number pf Total PTM-SNP	P-value
Phosphorylation	179	165489	0.021

Proteolytic Cleavage	2	1145	0.333

Disulfide bond	2	1253	0.373

S-palmitoyl Cysteine	1	1797	0.846

N-linked Glycosylation	7	9400	0.860

O-linked Glycosylation	3	5925	0.947

Acetylation	1	5579	0.997

Ubiquitination	4	15862	0.999

**Table 5:** Statistics of trait or disease-associated PTM-SNPs from GWAS catalogs

Disease	Number of SNP	Number of PTM-SNP	Ratio
Coronary heart disease	122	7	0.057

Diabetes Mellitus, Type 1	293	11	0.038

Lupus Erythematosus, Systemic	345	11	0.032

Diabetes Mellitus, Type 2	279	6	0.022

Macular Degeneration	661	11	0.017

Stroke	1490	17	0.011

Coronary Artery Disease	700	7	0.010

Heart Failure	1127	10	0.009

Cholesterol, LDL	831	7	0.008

Iron deficiency	961	7	0.007

### Case Study for Type 2 Diabetes associated PTM-SNP

Among the top-ranked diseases with disease-associated PTM-SNPs, T2D was selected for an in-depth analysis. T2D-associated PTM-SNPs were collected not only from GWAS catalog, GAD and dbGaP but also by mapping PTM-SNPs and 456 T2D-associated SNPs within the p-value threshold 1.0 × 10^-5 ^extracted from our previous study. Table 6 shows T2D-associated PTM-SNPs, including PTM name, p-value, gene name, and matching result with known T2D genes from public disease gene databases. In total, 6 T2D-associated PTM-SNPs are detected in 5 genes; PCSK1, C6orf57, WFS1, PPARG, and KCNJ11. Among them, WFS1, PPARG, and KCNJ11 are known T2D genes and were identified in T2D related functional modules such as glucose homeostasis (GO:0042593). PCSK1 is associated with obesity and diabetic nephropathy from previous GWASs and could be a T2D candidate gene.

**Table 6:** Type 2 diabetes-associated PTM-SNPs

SNP	PTM	p-value	Gene	Known T2D gene
6235	Phosphorylation	1.00E-26	PCSK1	

5219	Phosphorylation	7.00E-11	KCNJ11	O

5215	Phosphorylation	5.00E-11	KCNJ11	O

1801214	N-linked Glycosylation	3.00E-08	WFS1	O

1048886	Phosphorylation	3.00E-08	C6orf57	

1801282	Phosphorylation	2.00E-06	PPARG	O

One of T2D-associated PTM-SNPs, rs1801282, is located on PPARG which is a popular T2D gene and a T2D drug target for a thiazolidinedione and changed the Pro12 to the alanine. The SNP is reported T2D from previous study [30], however, the detailed correlation between the SNP and T2D have not been clearly known yet. According to PTM-SNP, rs1801282 may enhance the phosphorylation on Ser8 by CSNK2A1, prohibit the phosphorylation on Ser14 by MTOR or MAPK10, and also may affect the phosphorylation on Ser16. Not only PPARG but CSNK2A1, MAPK10, and MTOR are known as T2D genes and participate on parts of T2D associated pathways such as type II diabetes mellitus, insulin signaling pathway, and Wnt signaling pathway in KEGG.

The associations with T2D, body mass index (BMI), and weight of a Val66Met PTM-SNP (rs6265) located on BDNF are discovered from NHGRI GWAS catalogs and WTCCC T2D dataset analysis. The association between obesity and diabetes is discovered with rs6265 due to GWASs on BMI, weight, and T2D identified G allele as a risk allele and A allele as a non-risk allele. Adjacent site Thr62 of BDNF is predicted as a phosphorylation site and kinases for Thr62 are predicted as ARAF, RAF1, MAP3K2, and MAP3K4 by GPS2.0 and filtrated by STRING. RAF1 is known as a T2D gene and a member of insulin signaling pathway.

PTMs play crucial roles in biological mechanisms, and PTM-SNPs may influence PTM network regulation and have effects on associated diseases. From disease-associated PTM-SNPs, we can find disease-associated PTM networks regulated by PTM-SNPs that may provide insight into the mechanisms of disease. Our case studies show the possibility of using PTM-SNPs, combined with various types of biological information including known disease genes, drug targets, protein-protein interaction networks, and pathway networks, to identify mechanisms underlying disease. Novel disease-candidate genes can be found from disease-associated PTM networks assembled from the relations of PTM enzymes and PTM substrate proteins.

## Web interface

**Figure 2:**
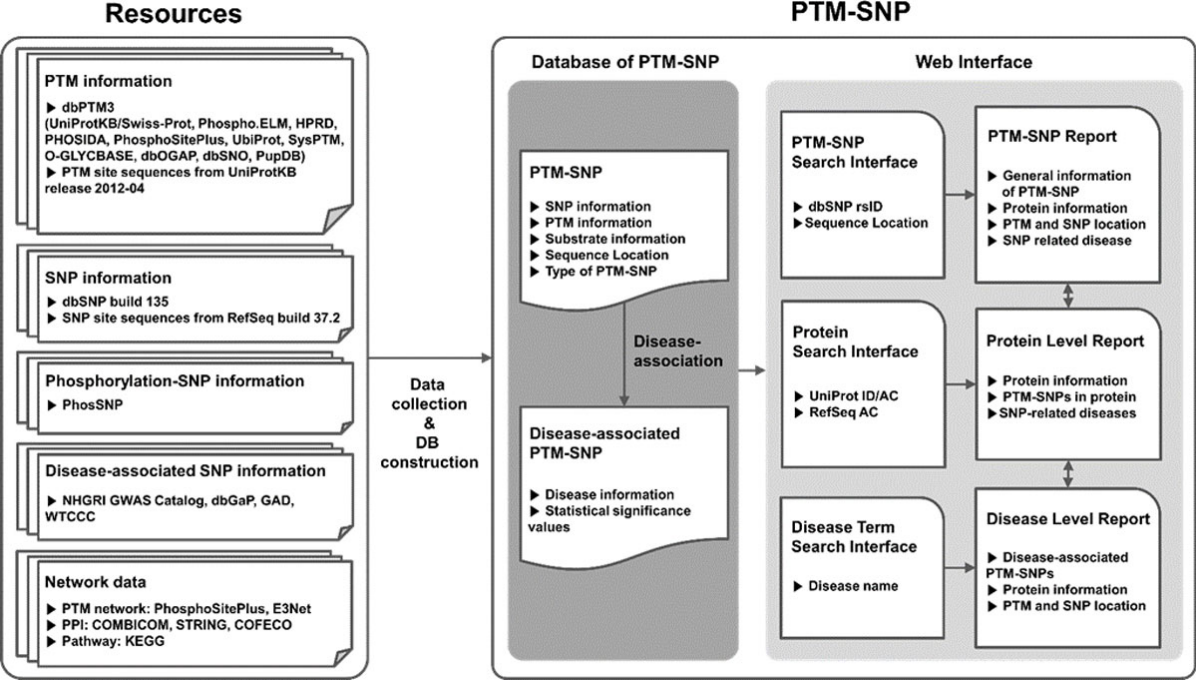
**Schematic illustration of data collection, analysis, and representation of PTM-SNPs**.

Collected PTM-SNPs and disease-associated PTM-SNPs are provided at the PTM-SNP website. The integrated PTM-SNP search interface allows users to search PTM-SNPs by dbSNP rs ID, RefSeq accession (AC), UniProt ID, or disease terms using the same search interface. Search results are provided with a PTM-SNP report, protein-level report or disease-level report, depend on the kind of the input search terms. When user input a dbSNP rs ID, the PTM-SNP report is displayed with the general information about the PTM-SNP including dbSNP rs ID, RefSeq AC, UniProt ID, residue of SNP, sequence location of PTM binding site, residue of PTM binding site, relative sequence location of PTM-SNP from PTM binding site, PTM name, the type of PTM-SNP, and the disease-association of the PTM-SNP including disease name and p-value if available. All columns can be easily sorted by clicking up/down arrows. In addition to the PTM-SNP information, the protein-level report provides all other PTM-SNPs that have been identified in the same substrate protein when user input a protein identifier such as RefSeq AC or UniProt ID. The disease-term search interface provides the disease-level report including disease-associated PTM-SNPs with disease name. Researchers can search the disease-associated PTM-SNPs easily using PTM-SNP web interface (Figure 2).

## Conclusion

In this study, we performed the investigation to associate PTMs, SNPs and disease using PTM-SNPs and disease-associated GWAS studies. PTM-SNP is a database based on dbSNP build 135, dbPTM3, and public GWAS databases of disease-associated SNPs that influence PTMs. In total, more than 50 kinds of PTMs were considered. PTM sites involved in signalling pathways such as phosphorylation, ubiquitination, and acetylation tended to have a greater proportion of PTM-SNPs than other PTM sites. Genes that contain disease-associated PTM-SNPs have been collected and matched against disease genes and drug targets to elucidate the effects of the SNPs and out analysis showed that PTM-SNP has higher disease-associations as compared with non-PTM-SNPs. We were also able to identify some of the mechanisms of disease-associated SNPs using PTM-SNP data. Researchers can match their own GWAS datasets with the PTM-SNP database to find PTM-related effects of the disease-associated SNPs easily by using the PTM-SNP website. We expect that provides an insight into unveiling important PTMs involved in causing diseases and finding novel disease markers.

## Competing interests

The authors declare that they have no competing interests.

## Authors' contributions

YK and CK designed and implemented the proposed database and wrote the manuscript. BM constructed web interface of PTM-SNP. GSY designed and directed this study, and reviewed the manuscript. All authors worked on and approved the final manuscript.

## Supplementary Material

Additional File 1 Statistics of whole PTM site in PTM-SNP based on their PTM types Click here for file

Additional File 2 Statistics of whole trait or disease-associated PTM-SNPs from GWAS catalogs Click here for file

## Declarations

Publication of this article has been funded by the Bio-Synergy Research Project (2012M3A9C4048759) of the Ministry of Science, ICT and Future Planning through the National Research Foundation (NRF), and by the KAIST Future Systems Healthcare Project from the Ministry of Science, ICT and Future Planning, and by the National Research Foundation of Korea (NRF) grant funded by the Korea government (MSIP) (No. 2010-0028631).

This article has been published as part of *BMC Medical Genomics *Volume 8 Supplement 2, 2015: Selected articles from the 4th Translational Bioinformatics Conference and the 8th International Conference on Systems Biology (TBC/ISB 2014). The full contents of the supplement are available online at http://www.biomedcentral.com/bmcmedgenomics/supplements/8/S2.
